# Hints to Young Practitioners on the Application of Certain Bandages, &c.

**Published:** 1855-05

**Authors:** B. E. Dodson

**Affiliations:** Morris, Illinois


ART. IV.—*Hints to Young Practitioners on the application of Certain Bandages, &c. &c.* By B. E. DODSON, M. D., of Morris, Illinois.

In the treatment of either the superior or inferior extremities, for the various injuries to those parts, fractures, dislocations, varicose-veins, ulcers of the leg, &c. &c., the practitioner is called upon to adjust bandages, and though in all the works on surgery he will find ample and definite directions for the manner of adjustment, &c.,yet, he will sometimes encounter difficulties attributable to what he would (*without reflection and experience*,) consider very minor causes.

I have now in my mind's eye, some half a dozen cases, where the patients have lost nearly all use, either of their hand, or foot, and the cause more to be imputed to injudiciously applied bandages, than to the original cause of their adjustment, and some of these too, from the hands of surgeons of considerable eminence.

The error to which I allude, is in commencing the adjustment of the bandage too far from the extremity of the limbs, consequently impeding the circulation at the extremity, and thereby inducing œdema, local inflammation, &c. &c., retarding the natural healing process, and finally a relaxation of muscular fibre, and a weakened, relaxed, debilitated, decayed limb for life. If these worst results are not realized, the patient is at least subjected to quite unnecessary pain, by those "*partial bandages*" which I have often relieved, by taking off the bandage, and commencing the adjustment at the extremity of the limb, thereby affording almost instantaneous relief from needless real affliction.

I am now treating an inveterate case of "Sore Shin" with caries of the bones, &c. He has been under treatment for six years, and I have no hopes of affording any permanent relief. The leg has been bandaged for years, *immediately* over the ulcer, until the leg was swelled above and below to nearly twice its normal size, and the veins are varicose, the patient very lame, and suffers great pain. I adjusted a bandage commencing "*at the extremity*," continued to the knee, three months since—the leg is assuming its normal shape,—he walks without limping—the ulcer discharges less, and a more healthy discharge—he suffers slight pain—says he is getting well—attributes the change to the little medication that has been used, &c. &c. His hopes, will not be realized, but *the hint*, may be worth something to some one about to "adjust a bandage."

MORRIS, ILL., March 20th, 1855.

